# Callus-mediated plant regeneration and CRISPR/Cas9-targeted mutagenesis in *Oenanthe javanica*

**DOI:** 10.1093/hr/uhaf327

**Published:** 2025-11-27

**Authors:** Kai Feng, Cheng Yao, Hui Lv, Zhiyuan Yang, Jialu Liu, Ziqi Zhou, Nan Sun, Shuping Zhao, Peng Wu, Aisheng Xiong, Liangjun Li

**Affiliations:** College of Horticulture and Landscape Architecture, Yangzhou University, Yangzhou 225009, China; College of Horticulture and Landscape Architecture, Yangzhou University, Yangzhou 225009, China; College of Horticulture and Landscape Architecture, Yangzhou University, Yangzhou 225009, China; College of Horticulture and Landscape Architecture, Yangzhou University, Yangzhou 225009, China; College of Horticulture and Landscape Architecture, Yangzhou University, Yangzhou 225009, China; College of Horticulture and Landscape Architecture, Yangzhou University, Yangzhou 225009, China; College of Horticulture and Landscape Architecture, Yangzhou University, Yangzhou 225009, China; College of Horticulture and Landscape Architecture, Yangzhou University, Yangzhou 225009, China; College of Horticulture and Landscape Architecture, Yangzhou University, Yangzhou 225009, China; State Key Laboratory of Crop Genetics & Germplasm Enhancement and Utilization, Ministry of Agriculture and Rural Affairs Key Laboratory of Biology and Germplasm Enhancement of Horticultural Crops in East China, College of Horticulture, Nanjing Agricultural University, Nanjing 210095, China; College of Horticulture and Landscape Architecture, Yangzhou University, Yangzhou 225009, China; Joint International Research Laboratory of Agriculture and Agri-Product Safety of Ministry of Education of China, Yangzhou University, Yangzhou 225009, China

Dear Editor,

*Oenanthe javanica* (Blume) DC., a perennial aquatic herb in the Apiaceae family, is widely cultivated in East Asian countries [1]. *Oenanthe javanica* is a medicinal and edible plant, which has high economic value and is popular for its distinctive aroma and crisp texture. Rich nutrients and pharmacological substances confer on *O. javanica* the therapeutic potentials, such as calming the liver, reducing blood pressure, preventing thrombosis, and anticancer effects [2]. Due to germination disorder and seed dormancy, *O. javanica* is commonly propagated through stem-cuttings in cultivation. Traditional asexual propagation has many disadvantages, such as high propagation costs, low reproductive coefficient, season dependence, and viral disease occurrence [3]. The long-term asexual propagation of *O. javanica* restricted the genetic diversity and germplasm innovation through traditional crossbreeding. The emergence of plant tissue culture and genetic transformation provides the efficient strategy to address these challenges [4].

Plant tissue culture technology has been developed on the basis of the totipotency of plant cells, including micropropagation, adventitious shoot regeneration, and somatic embryogenesis. Varieties, types of explants, disinfection processes, and the composition of the medium are the main factors influencing the plant tissue culture. The composition of the medium mainly includes macro elements, trace elements, iron salts, sucrose, vitamin, and plant growth hormone. Efficient tissue culture technology is an important basis for constructing a plant genetic transformation system. Genetic transformation can be used for the overexpression and silencing expression of functional genes in plants. The CRISPR/Cas (clustered regularly interspaced short palindromic repeat/CRISPR-associated protein) system for genome editing has been widely used in precise engineering of genomes and crop breeding [5]. *PDS* gene is a key gene in carotenoid biosynthesis, responsible for converting the colorless compound phytoene into the colored compound ζ-carotene. It plays an important role in photosynthesis and pigment biosynthesis in plants, and disruption of its function leads to albinism. Recently, the T2T genome of *O. javanica* has been published, which provides important genetic information for the gene editing in *O. javanica* [6].

Here, the callus-mediated regeneration and CRISPR/Cas9-targeted mutagenesis system in *O. javanica* were established. The vigorous axillary buds of *O. javanica* were sterilized and cultivated to obtain the aseptic seedlings. The petioles, leaf blades, and roots of *O. javanica* were selected as different explant types to evaluate the callus induction efficiency. The different kinds of explants were cut into ~5-mm-sized fragments and cultivated under dark conditions. Based on the quantitative comparisons of callus induction efficiency of different explants, petioles were investigated to be the most suitable explants for callus induction in *O. javanica* (Fig. S1). Similarly, petioles were also used as the suitable explant in other Apiaceae species, including celery [7] and carrot [8]. The callus induction efficiency is usually affected by many factors, such as plant growth regulators (PGRs) and basic culture medium. Murashige & Skoog (MS) medium and Gamborg B5 medium were selected as the basic mediums for callus induction of *O. javanica*. The petioles of aseptic seedling were used as explants and inoculated on MS and B5 medium supplemented with different concentrations of 2,4-D, NAA and 6-BA. Based on the comprehensive analysis of all treatments, B5 culture medium supplemented with 0.5 mg/l of 2,4-D and 1.0 mg/l of 6-BA showed the highest induction rates and better callus growth status than other treatments (Tables S1–S4). Notably, B5 culture medium supplemented with 1.0 mg/l of 2,4-D and 0.5 mg/l of 6-BA was the most suitable treatment for callus proliferation (Tables S5 and S6). MS basic medium supplemented with 0.5 mg/l of NAA and 2.0 mg/l of 6-BA was the optimum medium for callus differentiation of *O. javanica* (Table S7). Hence, the efficient callus-mediated regeneration system of *O. javanica* was established (Fig. 1A and Fig. S2A).

**Figure 1:**
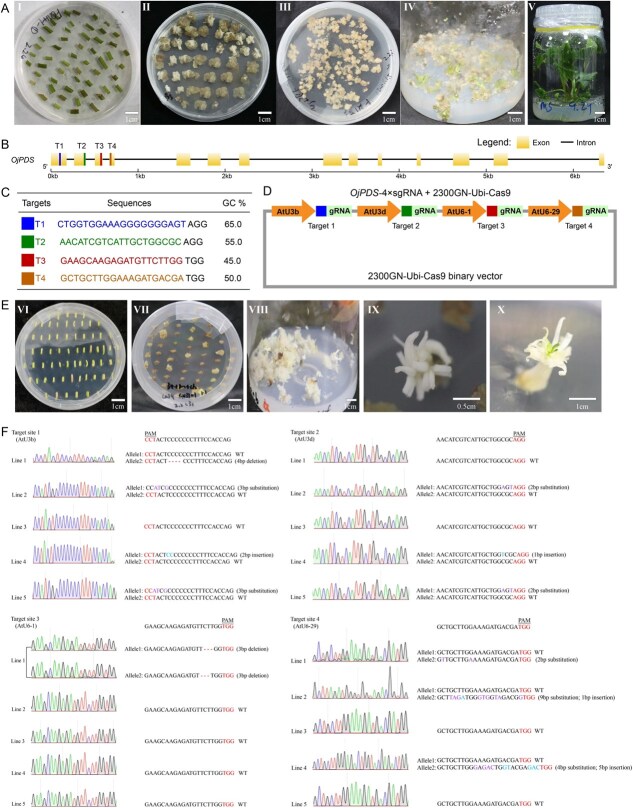
Callus-mediated plant regeneration and CRISPR/Cas9-mediated targeted mutagenesis of *Oenanthe javanica phytoene desaturase* (*OjPDS*) gene. (A) Processes of callus induction and plant regeneration of *O. javanica*. I, Petiole as explant; II, Calli induction; III, Calli proliferation; IV, Calli differentiation; V, Regenerated plant. (B) The genomic structure analysis and targets selection of *OjPDS* gene. T1, T2, T3, and T4 represent the positions of the four target sites individually located in the first four exons, respectively. (C) Sequences of four target sites for CRISPR/Cas9-mediated targeted mutagenesis of *OjPDS*. (D) Schematic map of *OjPDS*-2300GN-Ubi-Cas9 vector for CRISPR/Cas9-mediated targeted mutagenesis in *O. javanica*. (E) Processes of CRISPR/Cas9-mediated targeted mutagenesis of *OjPDS* in *O. javanica*. VI, Petiole as explant for targeted mutagenesis; VII, Calli induction for targeted mutagenesis; VII, Calli proliferation for targeted mutagenesis; IX, gene edited plant; X, chimeric plant. (F) Mutation detection of gene edited plants (Lines 1–5). The sequencing chromatograms of four target sites regions. The PAM sequence is highlighted.

Loss-function of *phytoene desaturase* (*PDS*) gene usually lead to the albino phenotype, which is widely used as visual target gene for evaluating the efficiency of gene editing in plants. To obtain the *PDS* homologous alleles, the sequence of *DcPDS* (GenBank accession XM_017385289.1) was used to conduct the BLAST alignment in the genome of *O. javanica* [6]. One single-copy *PDS* gene, *Oj17G000040.1*, was identified from *O. javanica* and designated as *OjPDS* (Figs S3 and S4). The *OjPDS* gene was cloned from *O. javanica* cv. ‘Fuqin No.1’ using the specific primers OjPDS-F and OjPDS-R (Table S8). OjPDS protein contains the phytoene–desat domain, which belongs to the phytoene dehydrogenase family and was involved in the carotene biosynthesis (Fig. S5). To ensure the precise editing of *OjPDS* gene, four target sites (T1–T4) individually located in the first four exons were designed using CRISPR-GE tool (Fig. 1B and C) [9]. Four Arabidopsis promoters (AtU3b, AtU3d, AtU6-1, and AtU6-29) were selected to individually drive the expression of four sgRNA cassette containing T1–T4 targets to improve the gene editing efficiency (Fig. S6). Then, the four sgRNA expression cassettes were constructed into the modified pYLCRISPR/Cas9Pubi-H vector, and the recombinant *OjPDS*-2300GN-Ubi-Cas9 binary vector was transformed into the *Agrobacterium tumefaciens* GV3101 for genetic transformation assay (Fig. 1D) [7].

Based on our optimized callus-mediated regeneration system, 603 explants were infected with *A. tumefaciens* GV3101 containing *OjPDS*-2300GN-Ubi-Cas9. To screen the positive transformed callus, the transformed explants were cultivated on B5 culture medium supplemented with 0.5 mg/l of 2,4-D, 1.0 mg/l of 6-BA, 50 mg/l of kanamycin, 100 mg/l of l-serine and 300 mg/l of carbenicillin. Finally, a total of 14 lines of *O. javanica* plants were regenerated from the callus *via* somatic embryogenesis pathway, including albino, green, and chimeric plants (Fig. 1E and Fig. S2B). To determine the efficiency of CRISPR/Cas9-mediated targeted mutagenesis, the genomic DNA was extracted from the positive plants using CTAB method. The genomic sequences harboring four target sites were amplified from the extracted DNA using PDS-detection-F and PDS-detection-R primers (Table S8). The PCR products were direct sequenced or cloned into the pCE2 TA/Blunt-Zero vector followed by Sanger sequencing. The decoded sequences indicated that ten lines of *O. javanica* were edited, indicating the CRISPR/Cas9-mediated targeted mutagenesis was constructed with the efficiency of 1.7%. Multiple mutation types were detected from the gene edited *O. javanica* plants, including base deletion, base insertion, base substitution and combination mutagenesis. The mutation efficiency at T1 (targeted by AtU3b-driven sgRNA) was 90%, including two homozygous mutations and four biallelic mutations, while the remaining were heterozygous mutations. At T2 (targeted by AtU3d-driven sgRNA), the mutation efficiency was 70%, consisting of three homozygous mutations and four heterozygous mutations. The mutation efficiency at T3 (targeted by AtU6-1-driven sgRNA) was 20%, including one heterozygous mutation and one biallelic mutation. The mutation efficiency at T4 (targeted by AtU6–29-driven sgRNA) was the same as that at T2, including six heterozygous mutations and one biallelic mutation. The mutation efficiency at T1, T2, and T4 was significantly higher than at T3 (Fig. 1F and Fig. S7). Homozygous mutations were observed at the T1 and T2 targets, while no such mutations were detected at T3 and T4. This indicates that the observed differences in mutation efficiency among T1, T2, T3, and T4 are related to promoter type. Furthermore, the mutation efficiency at T1 was higher than that at T2. The mutation efficiency at T1 was driven by AtU3b promoter, which has been widely used in various crops for gene editing [10]. Among the gene-edited plants, four lines were exhibited as heterozygotes, with a frequency of 40%. The frequency of chimeras and homozygous phenotypes were calculated as 20% and 40%, respectively.

In conclusion, this study constructed the efficient callus-mediated plant regeneration by optimizing the explant type, basic culture medium, varieties and proportion of PGRs. This study also provided the first report of CRISPR/Cas9-mediated targeted mutagenesis in *O. javanica*. These results will have tremendous application prospects in functional genes verification and innovation of excellent germplasm through molecular breeding in *O. javanica*.

## Data Availability

The supplementary methods and data supporting the conclusions of this article are available on the Figshare database under the accession https://doi.org/10.6084/m9.figshare.30617534.v2.

## References

[1] Wang XJ, Luo Q, Li T. et al. Origin, evolution, breeding, and omics of Apiaceae: a family of vegetables and medicinal plants. Hortic Res. 2022;9:uhac076 38239769 10.1093/hr/uhac076 PMC10795576

[2] Lu CL, Li XF. A review of *Oenanthe javanica* (Blume) DC. as traditional medicinal plant and its therapeutic potential. Evid Based Complement Alternat Med. 2019;2019:1–17 10.1155/2019/6495819 PMC6463588 31057651

[3] He Z, Sheng SY, Wang LQ. et al. Cucumber mosaic virus-induced gene and microRNA silencing in water dropwort (*Oenanthe javanica* (Blume) DC). Plant Methods. 2024;20:6 38212839 10.1186/s13007-023-01129-4 PMC10782793

[4] Zhang X, Xu G, Cheng C. et al. Establishment of an Agrobacterium-mediated genetic transformation and CRISPR/Cas9-mediated targeted mutagenesis in Hemp (*Cannabis sativa* L.). Plant Biotechnol J. 2021;19:1979–87 33960612 10.1111/pbi.13611 PMC8486249

[5] Wolabu TW, Mahmood K, Jerez IT. et al. Multiplex CRISPR/Cas9-mediated mutagenesis of alfalfa *FLOWERING LOCUS Ta1* (*MsFTa1*) leads to delayed flowering time with improved forage biomass yield and quality. Plant Biotechnol J. 2023;21:1383–92 36964962 10.1111/pbi.14042 PMC10281603

[6] Feng K, Liu JL, Sun N. et al. Telomere-to-telomere genome assembly reveals insights into the adaptive evolution of herbivore-defense mediated by volatile terpenoids in *Oenanthe javanica*. Plant Biotechnol J. 2025;23:2346–57 40112135 10.1111/pbi.70062 PMC12120883

[7] Liu JX, Li T, Wang H. et al. CRISPR/Cas9-mediated precise targeted mutagenesis of phytoene desaturase in celery. Hortic Res. 2022;9:uhac162 36204201 10.1093/hr/uhac162 PMC9531335

[8] Xu ZS, Feng K, Xiong AS. CRISPR/Cas9-mediated multiply targeted mutagenesis in orange and purple carrot plants. Mol Biotechnol. 2019;61:191–9 30644027 10.1007/s12033-018-00150-6

[9] Xie XR, Ma XL, Zhu QL. et al. CRISPR-GE: a convenient software toolkit for CRISPR-based genome editing. Mol Plant. 2017;10:1246–9 28624544 10.1016/j.molp.2017.06.004

[10] Hu N, Xian Z, Li N. et al. Rapid and user-friendly open-source CRISPR/Cas9 system for single- or multi-site editing of tomato genome. Hortic Res. 2019;6:7 30603093 10.1038/s41438-018-0082-6 PMC6312546

